# Special Issue “The State of the Art in Endodontics”

**DOI:** 10.3390/jcm11092329

**Published:** 2022-04-22

**Authors:** Alfredo Iandolo, Alessandra Amato, Dina Abdellatif, Giuseppe Pantaleo, Massimo Amato

**Affiliations:** 1 Department of Conservative and Endodontics, Faculty of Dentistry, University of Salerno, 84084 Salerno, Italy; aale.amato@gmail.com (A.A.); giuseppepantaleo88@gmail.com (G.P.); maxamato1@gmail.com (M.A.); 2 Department of Endodontics, Faculty of Dentistry, University of Alexandria, Alexandria 21545, Egypt; dinaabdellatif81@gmail.com

Currently, the term “modern endodontics” is used more often due to contemporary applied science and original materials that have been developed in recent years. Various instruments and devices were developed to simplify and improve our endodontic treatments. For instance, these include operating microscopes, ultrasonic devices, different lasers, modified alloys for rotating NI-Ti files, powerful irrigation strategies, the latest irrigant solutions, newly developed materials for filling root canals, 3D (three dimensional) radiography, and several more [1]. Furthermore, difficult root canal treatments can be performed safely when these advanced techniques are employed, consequently ensuring adequate therapy for patients and saving teeth that would otherwise be condemned for extraction. General practitioners and endodontists, who are equally important, should be aware of and apply these advanced techniques in their daily work.

The current Special Issue, “The State of the Art in Endodontics”, in the *Journal of Clinical Medicine*, is dedicated to collecting high-quality scientific contributions that mainly focus on modern technologies and protocols.

Presently, with the introduction of 3D radiography and CBCT in endodontics, more precise correct diagnoses can be achieved. For example, it is possible to anticipate complex anatomies, identify root fractures, make a differential diagnosis between external and internal resorptions, and identify small periapical lesions that are not perceptible with traditional radiology [1,2]. All of these aspects can improve the prognosis of the treatment to be carried out.

After reaching a correct diagnosis, endodontic treatment begins by the preparation of an access cavity. In recent years, the concept of minimally invasive endodontics has been advancing progressively. This concept begins with access cavities.

By creating conservative cavities, it is possible to save more dental tissue and avoid the risk of fracture [3].

Once the access cavity is prepared and all root canals have been identified, the subsequent shaping phase can be begun.

Two studies in the current Special Issue studied the shaping phase during endodontic treatment [4,5]. Nickel–titanium (NiTi) endodontic rotary files allow clinicians to maintain the original anatomy of root canals, especially in curved canals. Consequently, the possibility of conceivable mishaps during the mechanical preparation of a root canal system is diminished. The innovation of the heat treatment process during the manufacturing of NI-Ti files allows for modifications in the physical properties of the NiTi rotating instrument. For instance, this treatment increases cyclic fatigue resistance and helps the files to conform to diverse curves and angles in a root canal. Research has illustrated the utilization of a single rotary instrument in a reciprocation action when treating primary teeth and reported considerable advantages in pedodontics. For example, the therapy time was reduced, the liability for iatrogenic mistakes was also diminished, and cross-contamination between patients was prevented [6,7].

It has been reported that the shaping phase alone, regardless of the file used, cannot reach the whole of the complex endodontic space. Manual and rotating files can only be used in the central portion of the root canal. Files cannot reach lateral anatomies such as isthmuses, lateral canals, loops, deltas, and similar. For this reason, the cleaning phase is important and is considered a fundamental step to adequately eliminating bacteria and necrotic tissue [8].

Furthermore, minimally invasive shaping protocols in the form of using rotary files that are small in size and taper promote more efficient irrigation protocols and safer, more conservative endodontic treatments [9]. 

Sodium hypochlorite (NaOCl) is considered the most common irrigant used during the cleaning phase owing to its high tissue dissolution ability and prominent antimicrobial action [10]. 

Several techniques can be applied to utilize the action and effect of NaoCl. A recently introduced technique, internal heating, combined with ultrasonic activation, can also achieve excellent results in the case of conservative shaping [8,11].

Numerous additional antimicrobial solutions were suggested for use in root canal chemical cleaning, such as benzalkonium chloride (BAK) and chlorhexidine (CHX) [12,13].

In our current Special Issue, new research evaluated the antibacterial effect and depth of penetration of a chitosan nanodroplet (ND) solution packed with benzalkonium chloride (BAK) inside dentinal tubules [14]. The study showed that BAK induces structural disorganization, the loss of cytoplasmic membrane integrity, and has damaging impacts on microorganisms [15]. Additionally, when BAK is used in solution concentrated up to 5%, it is believed to offer antibacterial effects and durable outcomes as it inhibits the proteases of microorganisms [15].

In this research, the NaOCl solution showed the highest antimicrobial efficacy, but nanodroplets with BAK seemed to have an identical effect to CHX, with a high depth of efficacy.

After finishing the shaping and cleaning step, the obturation phase occurs next in the endodontic space.

In recent years, new sealers with excellent properties, such as biosealers, have also been developed, in addition to the invention of new techniques for use in the obturation phase [16]. The major features of these new sealers are their increased PH, greater antibacterial activity, decreased setting time, biocompatibility, and micro-expansion in the root canal.

In the current Special Issue [17], a new technique was evaluated that employs ultrasonic tips to apply sealer in the root canal. 

Ultrasonic devices have been effectively employed in the field of endodontics in recent decades for most of the endodontic steps, including root canal obturation [18]. 

Many studies showed that the application of ultrasonic energy on sealers during the root canal filling procedure can boost the sealer’s penetration inside the dentinal tubules and enhance the boundary connection between the obturation material and the root canal wall [17]. In addition, ultrasonic energy can rearrange the sealer particles and eradicate the trapped air, hence decreasing the porosity.

Utilizing micro-CT analysis, the study evaluated the effect of direct ultrasonic activation on the porosity diffusion in biosealer in root canal filling. Within the limits of this in vitro study, they concluded that none of the obturation procedures could offer pore-free endodontic obturation in the apical 5 mm. 

After the completion of the obturation phase, up to 40% of patients may report postoperative pain [19].

Postoperative pain can remain for some time after the treatment and can be intense according to multiple prognostic aspects. The factor that corresponds the most to postoperative pain is the obturation technique; this can be in the form of a cold lateral, single cone, or warm vertical compaction technique. Moreover, the sealer was found to play an important role in postoperative pain, with the most traditionally used being resin-based or zinc-oxide eugenol sealers [19].

In this Special Issue, using a systematic review and meta-analysis, Mekhdieva et al. reported the impact of using the biosealer filling technique in comparison to conventional obturation processes on postoperative pain in adult patients following endodontic treatment [20].

Mekhdieva et al. proposed that the biosealer obturation method may have a positive effect on postoperative pain. Concurrently, it was reported that many factors affected the flare-up of pain, for example, if analgesics were administered, the pulp status, and the number of visits when using biosealer compared with resin-based sealer. Nevertheless, additional well-designed clinical studies are warranted to augment their results due to several restrictions in their analyses.

In conclusion, the knowledge and the application of modern technologies and the continuous search for and development of new techniques and endodontic materials are crucial to making root canal treatment safer and more efficient.

As the Guest Editors, we would like to sincerely appreciate and thank the reviewers for their insightful remarks and the *JCM* team’s support. Ultimately, we heartily thank all of the contributing authors for their valuable input.
